# Role of the autophagy-related marker LC3 expression in hepatocellular carcinoma: a meta-analysis

**DOI:** 10.1007/s00432-020-03174-1

**Published:** 2020-03-10

**Authors:** Yu-Chen Meng, Xiao-Li Lou, Li-Yuan Yang, Dong Li, Yan-Qiang Hou

**Affiliations:** 1 Department of Central Laboratory, Songjiang Hospital Affiliated First People’s Hospital, Shanghai Jiao Tong University, 746 Zhongshan Road, Shanghai, 201600 China; 2 Department of Laboratory Medicine, Tongji Hospital of Tongji University School of Medicine, 389 Xincun Road, Shanghai, 200065 China

**Keywords:** LC3, Hepatocellular carcinoma, Meta-analysis, Prognosis

## Abstract

**Background:**

Microtubule-associated protein 1 light chain 3 (LC3), an autophagic gene, has been reported as a vital marker for many diseases and cancers. However, the role of LC3 in hepatocellular carcinoma (HCC) was not still investigated. Therefore, we conducted a meta-analysis to examine the association of LC3 with its clinicopathological and prognostic in HCC.

**Methods:**

We consulted the PubMed, Cochrane Library, Web of Science, EMBASE, China National Knowledge Infrastructure and Wan Fang databases for published studies on LC3 in HCC. Newcastle–Ottawa scale was used to screen the quality of the literature. The statistical analysis was calculated by STATA 14.2.

**Results:**

Of the 1329 titles identified, 10 articles involving 949 patients in HCC were included in this meta-analysis. The results of our study show that increased LC3 expression is related to size of tumor, but not to gender, age, number of tumor, liver cirrhosis, HBsAg, TNM stage, alpha fetoprotein, vascular invasion and histological grade. Positive LC3 expression was associated with overall survival by pooled hazard ratio.

**Conclusions:**

This meta-analysis indicated that positive LC3 expression was related to size of tumor, and could predict prognosis in human hepatocellular carcinoma.

## Introduction

Hepatocellular carcinoma (HCC) is the third leading cause of cancer-related death and the fifth most common incidence rate (Yao et al. 2019), and it has a poor prognosis (Liang et al. 2018). About 800,000 of new cases of HCC in each year were examined worldwide, People’s Republic of China count more than 50% of these cases (Gingold et al. 2018). Currently, HCC was treated with surgery, chemotherapy, radiotherapy and sorafenib-targeted drug therapy (Hartke et al. 2017; Boyvat 2017). Although the treatment measures have made a great progress recently, the clinical cure rate is still poor, especially number of patients miss the surgery because of the lack of early detection and treatment (Grandhi et al. 2016). Therefore, developing effective early predictive marker is necessary to HCC.

Autophagy is an intracellular catabolic process which contributes to homeostasis, differentiation, recycling of damaged organelles or long-lived proteins (Lee and Jang 2015; Han et al. 2014). Increasing evidences have demonstrated that autophagy is associated with tumor development and progression (Lv et al. 2019). Several proteins are involved in the human autophagic pathway, the microtubule-associated protein 1 light chain 3 (LC3) and Beclin-1 play an important role which have been reported to associate with the physiology and pathogenesis of human liver disease (Chih et al. 2017).

LC3, the mammalian homolog of the yeast Atg8p, is considered as the crucial component of autophagosomes (Maruyama et al. 2014). It includes three isoforms of LC3A, LC3B, LC3C, of which LC3B is associated with autophagy levels (Wu et al. 2014a). LC3 has been reported in several tumors (including ovarian cancer (Shen et al. 2008), brain cancer (Ghavami et al. 2014), colorectal cancer (Wu et al. 2015), prostate cancer (Zhang et al. 2017), breast cancer (Jiang et al. 2017), melanoma (Segala et al. 2017). A meta-analysis of LC3 and ovarian cancer has shown that LC3 expression is associated with FIGO stage (Zhao et al. 2017). LC3 expression has also been found to show controversial results in the clinicopathological features in HCC patients (Lee et al. 2013; Bao et al. 2014; Ding et al. 2008). Wu et al. (2015) have indicated that LC3 expression is significantly associated with vascular invasion; high LC3 expression had indicated a poor overall survival (OS). However, Wu et al. (2014b) have found that LC3 expression is significantly correlated with male gender, large tumor size, tumor stage and worse relapse-free and OS. Chih et al. (2017) have demonstrated that the absence of LC3 expression is strongly predictive of immediate mortality for HCC. Hence, we conducted a meta-analysis to explore the relationship between LC3 expression and its clinicopathological characteristics and prognostic value in HCC.

## Methods

### Search strategy

A literature search of the PubMed, Web of Science, Cochrane Library, EMBASE, Chinese China National Knowledge Infrastructure (CNKI) and Chinese WanFang was performed to English and Chinese articles published before September 2019 using combinations of the following search terms: “LC3” or “microtubule-associated protein 1 light chain 3” or “Atg8p” and “hepatocellular carcinoma” or “HCC” or “liver cancer” or “hepatic tumor” or “liver tumor” or “hepatic cancer”.

### Eligibility criteria

Studies included in the meta-analysis had to meet the following inclusion criteria: (1) cohort or case–control design; (2) the patients were diagnosed with HCC and included clinicophathological or survival information; (3) LC3 expression was determined by immunohistochemistry (IHC). (4) The literature was published in English or Chinese and the full text was available.

The exclusion of studies were as follows: (1) case reports, reviews, letters, conference abstracts, simple commentaries, unpublished reports, (2) republished studies using the same dataset or patients, (3) the cohort or case–control design were unclear.

### Data extraction

All data were assessed by two reviewers (Xiaoli Lou and Liyuan Yang) who extracted data that include the first author’s name, publication year, sample size, country, type of cancer, detection method, patient characteristics, hazard ratio (HR) with 95% confidence interval (CI) for OS.

### Quality assessment

Quality assessment was assessed using the Newcastle–Ottawa Scale (NOS). The NOS total scores ranged from 0 to 9, and scores 1–3, 4–6, 7–9 were defined as low-, moderate-, or high-quality studies, respectively. The ten articles included in this meta-analysis were considered to have a high quality.

### Statistical analysis

The STATA software of version 14.2 (Stata Corporation, College Station, TX, USA) was performed in meta-analysis to assess the correlation between LC3 expression and clinicopathological features in HCC. Heterogeneity between the studies was tested using chi-squared test (*Q* test) and *I*^2^ test. There was no significant heterogeneity when *p* value (*Q* test) > 0.1 and *I*^2^ ≤ 50%, the fixed-effects model was chosen; otherwise, a random-effects model was used. To explore the sources of heterogeneity, we conducted subgroup analyses based on sample size, NOS score, area and average age. Publication bias was assessed by Begg’s test and funnel plot.

## Results

**Fig. 1:**
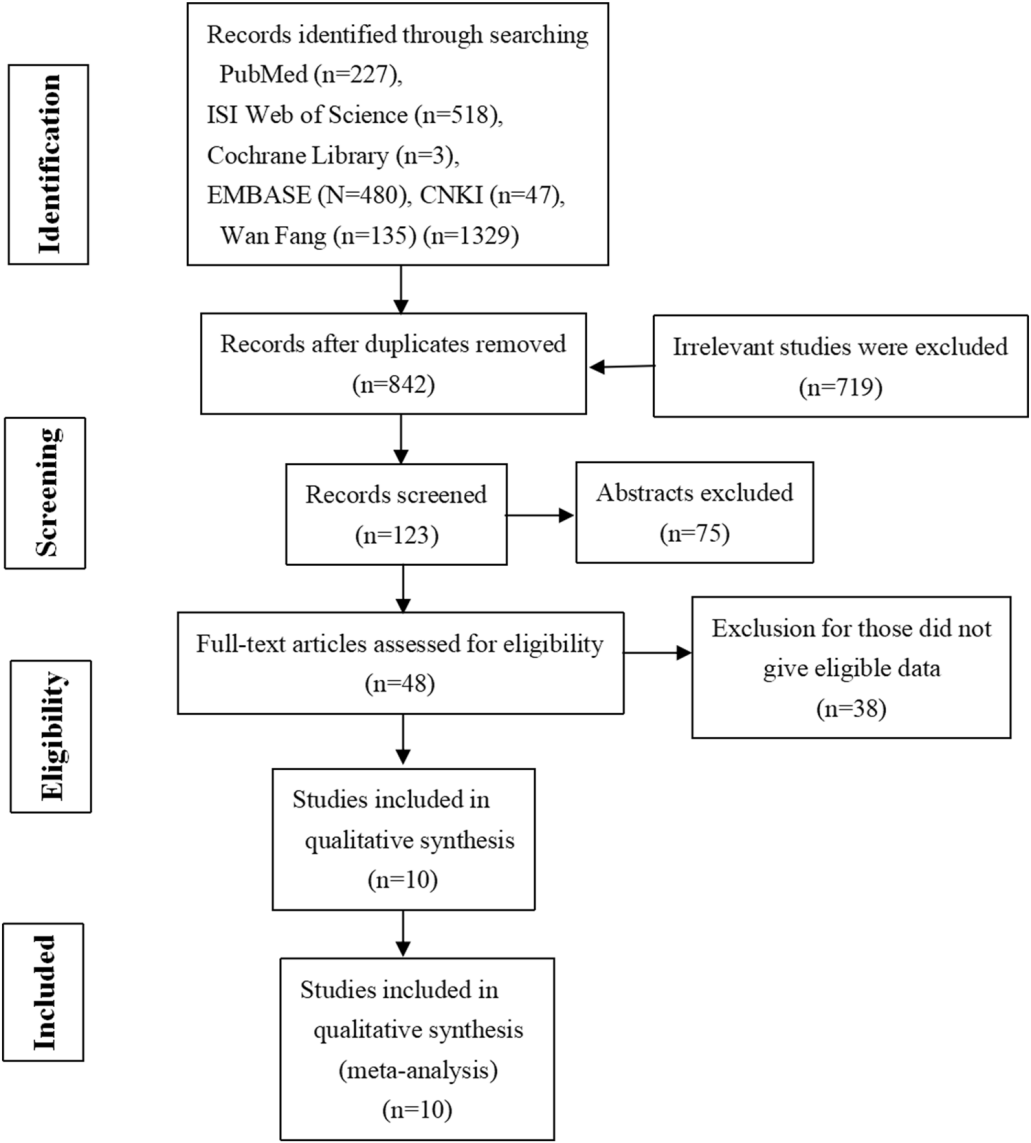
Flow chart of study selection

A total of 1329 relevant studies were identified from the database, and 1281 studies were excluded based on screening of duplication, titles, abstracts, non-LC3 relation (Fig. 1). Full text of 48 articles was retrieved and assessed for eligibility. Among 48 studies, 38 were excluded because of ineligible and duplicated data about LC3 expression. Finally, ten publications were eligible for this meta-analysis (Wu et al. 2014a, b,^ 2016^; Lee et al. 2013; Xi et al. 2013; Yu et al. 2018; Li et al. 2018; Zhao et al. 2016; Lu 2012; Song et al. 2014), all of which were cohort studies.

The characteristics of the included articles are summarized in Table 1. Four of the ten studies were written in English and six were in Chinese. These studies were published from 2012 to 2018 and a total of 949 patients with HCC were comprised. The number of patients ranged from 48 to 190; 6 studies include ≤ 100 patients, and 4 studies include > 100 patients. Among the ten studies, nine were from China, one from Korea. LC3 expression were investigated through IHC staining. The quality of each included article was evaluated by NOS. These scores of studies were all ≥ 6, indicating the high quality of included studies (Table 2).

**Table 1:** Characteristics of studies included in the meta-analysis

References	Year	Country	Tumor type	Number of patients	Sex Male (+/−) Female (+/−)	Age older (+/−) middle aged (+/−)	HbsAg yes (+/−) no (+/−)	Liver cirrhosis yes (+/−) no (+/−)	AFP positive (+/−) negative (+/−)	Tumor size > 5 cm (+/−) ≤ 5 cm (+/−)	Tumor number single (+/−) multiple (+/−)	Vascular invasion yes (+/−) no (+/−)	Histological grade low (+/−) and moderate, high (+/−)	TNM stage I, II (+/−) III, IV (+/−)	Method		Cutoff	OS data provide
Wu et al. (2014a)	2014	China	HCC	156	113/30	55/18	93/24	86/26	NA	60/11	79/22	33/2	109/28	52/20	IHC	4		Yes
					11/2	69/14	31/8	38/6		64/21	45/10	91/30	15/4	72/12				
Wu et al. (2014b)	2014	China	HCC	150	52/74	18/58	4773	50/86	NA	52/54	48/20	NA	104/32	35/80	IHC	50%		Yes
					3/21	37/92	8/22	5/9		3/44			55/95	20/15				
Xi et al. (2013)	2013	China	HCC	125	58/50	34/30	61/45	46/41	42/14	30/19	NA	37/19	29/10	30/32	IHC	1		Yes
					10/7	34/27	7/12	22/16	26/43	38/38		31/38	39/47	38/25				
Lee et al. (2013)	2013	Korea	HCC	190	32/126	23/74	27/110	20/76	9/47	16/60	NA	NA	11/25	17/63	IHC	5		No
					8/24	17/76	13/40	20/74	31/103	24/90			29/125	23/87				
Yu et al. (2018)	2018	China	HCC	56	30/10	26/10	28/10	NA	23/7	25/7	NA	NA	NA	NA	IHC	4		No
					9/7	13/7	11/7		16/10	14/10								
Li et al. (2018)	2018	China	HCC	49	25/13	19/14	25/11	NA	16/10	20/9	NA	8/2	24/12	25/14	IHC	4		No
					6/5	12/4	6/7		15/8	11/9		23/16	7/6	6/4				
Zhao et al. (2016)	2016	China	HCC	52	13/22	11/16	17/30	15/25	16/24	10/23	NA	0/1	19/15	17/14	IHC	4		No
					8/9	10/15	4/1	6/6	5/7	13/6		21/30	2/16	4/17				
Lu et al. (2012)	2012	China	HCC	62	20/26	14/13	21/28	NA	NA	NA	22/24	NA	11/3	22/33	IHC	1		No
					9/9	13/22	6/7				5/11		16/32	6/3				
Song et al. (2014)	2014	China	HCC	48	18/19	11/8	20/3	21/2	NA	9/12	NA	6/10	6/16	15/8	IHC	4		No
					5/6	17/12	22/3	24/1		14/13		17/15	17/9	8/17				
Wu et al. (2016)	2016	China	HCC	61	18/35	6/9	19/35	18/37	NA	18/27	NA	5/19	15/41	NA	IHC	1		No
					1/7	13/33	0/7	1/5		1/15		14/23	4/1					

HCC, hepatocelular carcinoma; AFP, alpha fetoprotein; NA, not applicable; IHC, immunohistochemistry; TNM stage, tumor-node-metastasis stage

**Table 2:** Newcastle–Ottawa scale for quality assessment

Study	Selection				Comparability	Outcome			Total score
	Exposed cohort	Non-exposed cohort	Ascertainment of exposure	Outcome of interest	Control for factor	Assessment of outcome	Follow-up long enough	Adequacy of follow-up	
Dong-Hao Wu	*	*	*	*	**	*	*	*	9
Wen-Yong Wu	*	*	*	*	**	*	*		8
Shao-Yan Xi	*	*	*	*	**	*	*	*	9
Yoo Jin Lee	*	*	*	*	**	*	*	*	9
Yu Hai	*	*	*	*	**	*			7
Li Fang	*	*	*	*	**	*			7
Ya-Tong Zhao	*	*	*	*	*	*	*	*	8
Yu-Dong Lu	*	*	*	*	**	*			7
Meng-Qi Song	*	*	*	*	*	*			6
Wen-Yong Wu	*	*	*	*	*	*	*	*	8

A study can be awarded one star for each numbered item within the selection and outcome categories. A maximum of two stars can be given for comparability. *1 points of score are added in quality assessment. **2 points of score are added in quality assessment. https://www.ohri.ca/programs/clinical_epidemiology/oxford.asp

### Meta-analysis of clinicopathological characteristics

**Fig. 2:**
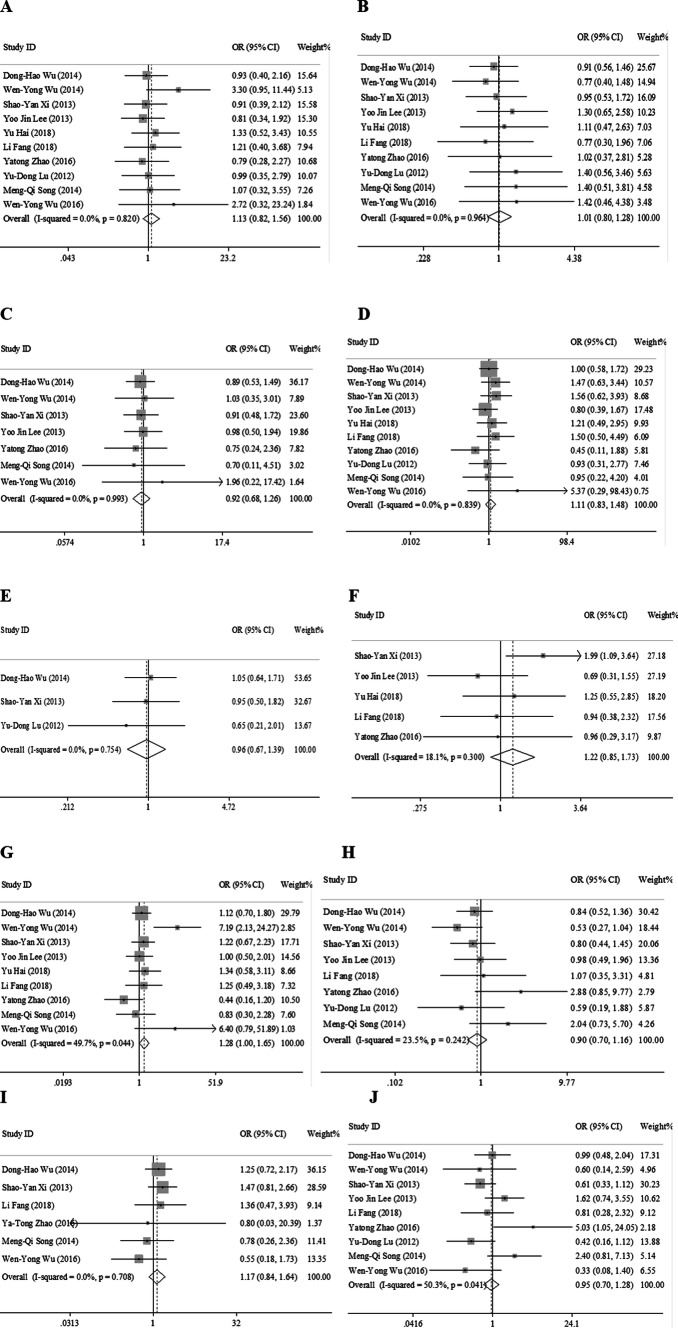
Forest plot of studies assessing the relationship between LC3 expression and **a** gender, ** b** age, ** c** liver cirrhosis, **d** HbsAg, ** e** tumor number,** f** AFP, ** g** tumor size, **h** TNM stage, ** i** vascular invasion, ** j** histological grade. * CI* confidence interval;* OR* odds ratio

In this study, we assessed the correlation between LC3 expression and the clinicopathological features of HCC. As shown in Fig. 2 and Table 3, positive LC3 expression was positively associated with tumor size (OR 1.28, 95% CI [1.00, 1.65], *p* = 0.050, fixed effect). However, the expression of LC3 was not associated with gender (OR 1.13, 95% CI [0.82, 1.56], *p* = 0.452, fixed effect), age (OR 1.01, 95% CI [0.80, 1.28], *p* = 0.920, fixed effect), number of tumors (OR 0.97, 95% CI [0.67, 1.39], *p* = 0.838, fixed effect), TNM stage (OR 0.90, 95% CI [0.70, 1.16], *p* = 0.424, fixed effect), alpha fetoprotein (AFP) (OR 1.22, 95% CI [0.85, 1.73], *p* = 0.276, fixed effect), vascular invasion (OR 1.17, 95% CI [0.84, 1.64], *p* = 0.357, fixed effect), liver cirrhosis (OR 0.92, 95% CI [0.68, 1.26], *p* = 0.618, fixed effect), HBsAg (OR 1.11, 95% CI [0.83, 1.48], *p* = 0.483, fixed effect) and histological grade (OR 0.95, 95% CI [0.70, 1.28], *p* = 0.718, random effect).

**Table 3:** LC3 clinicopathological features for HCC

Clinicopathological features	Number of studies	Number of patients	Pooled OR (95% CI)	Meta-regression * p* value	Heterogeneity		Model used
					*p* value	*I*^2^ (%)	
Gender	10	949	1.13 (0.82, 1.56)	0.452	0.820	0.0	Fixed
Age	10	949	1.01 (0.80, 1.28)	0.920	0.964	0.0	Fixed
HbsAg	10	949	1.11 (0.83, 1.48)	0.483	0.839	0.0	Fixed
AFP	5	472	1.22 (0.85, 1.73)	0.276	0.300	18.1	Fixed
Liver cirrhosis	7	782	0.92 (0.68, 1.26)	0.618	0.993	0.0	Fixed
Tumor size	9	887	1.28 (1.00, 1.65)	0.050	0.044	49.7	Fixed
Tumor number	3	343	0.97 (0.67, 1.39)	0.838	0.754	0.0	Fixed
Vascular invasion	6	491	1.17 (0.84, 1.64)	0.357	0.708	0.0	Fixed
Histological grade	9	893	0.95 (0.70, 1.28)	0.718	0.004	50.3	Random
TNM stage	8	783	0.90 (0.70, 1.16)	0.424	0.242	23.5	Fixed

,*Fixed* fixed-effects model, *Random* random-effects model

### Subgroup analysis

As shown in Table 4, to explore the potential sources of heterogeneity, the subgroup analysis for histological grade and tumor size was performed using the sample size, NOS score, area and mean age. When the classifications of subgroups were based on sample size, LC3 expression was related to tumor size (*n* > 100: OR 1.39, 95% CI [1.02, 1.88], *p* = 0.034, random effect) in the large sample size but not in the subgroup of small sample size. Heterogeneity of histological grade were in the small sample size subgroup (*n* ≤ 100, *I*^2^ = 66.9), and tumor size were in the large sample size subgroup (*n* > 100, *I*^2^ = 66.1). However, there was no heterogeneity in the tumor size with the small simple size and histological grade with the large simple size. The subgroup was classified by NOS score, LC3 expression was correlated with tumor size (*n* > 7: OR 1.33, 95% CI [1.00, 1.76], *p* = 0.051, random effect). Heterogeneity was in both subgroups of histological grade (*n* > 7, *I*^2^ = 52.9; *n* ≤ 7, *I*^2^ = 63.3). There was no heterogeneity in subgroup of tumor size (*n* ≤ 7, *I*^2^ = 0.0). On the basis of the area, the LC3 expression was connected with tumor size (midland: OR 1.73, 95% CI [1.04, 2.88], *p* = 0.035, random effect) and histological grade (north: OR 0.94, 95% CI [0.51, 1.75], *p* = 0.035, random effect; midland: OR 1.51, 95% CI [0.80, 2.86], *p* = 0.033, random effect). There was no heterogeneity in southern subgroup of tumor size and histological grade. Subgroup analysis based on mean age showed that expression of LC3 was associated with tumor size (age ≥ 55: OR 1.81, 95% CI [1.22, 2.70], *p* = 0.003, random effect). Heterogeneity was observed in tumor size subgroup (age ≥ 55, *I*^2^ = 61.0) and histological grade subgroup (age < 55, *I*^2^ = 66). Results of subgroups analysis revealed that the sample size, NOS score, area and mean age score most likely caused heterogeneity in tumor size and histological grade.

**Table 4:** Subgroup analysis of histological grade and tumor size by sample size, NOS score, area and average age

Subgroups	Number of studies	Number of patients	Pooled OR (95% CI)	*p* value	PHet	*I*^2^ (%)	Model used
*Tumor size*							
Sample							
*n* > 100	4	621	1.39 (1.02, 1.88)	0.034	0.031	66.1	Random
*n* ≤ 100	5	218	1.09 (0.70, 1.69)	0.697	0.166	38.2	Fixed
NOS score							
> 7	6	734	1.33 (1.00, 1.76)	0.051	0.009	67.6	Random
≤ 7	3	105	1.15 (0.68, 1.95)	0.610	0.753	0.0	Fixed
Area							
North	2	246	1.13 (0.66, 1.92)	0.662	0.600	0.0	Fixed
Midland	4	311	1.73 (1.04, 2.88)	0.035	0.001	81.2	Random
South	3	330	1.17 (0.83, 1.66)	0.364	0.964	0.0	Fixed
Average age							
≥ 55	5	506	1.81 (1.22, 2.70)	0.003	0.036	61.0	Random
< 55	4	381	1.01 (0.73, 1.40)	0.967	0.340	10.6	Fixed
*Histological grade*							
Sample							
*n* > 100	4	621	0.88 (0.60, 1.31)	0.536	0.251	26.8	Fixed
*n* ≤ 100	5	272	1.05 (0.64, 1.71)	0.843	0.017	66.9	Random
NOS score							
> 7	6	734	0.96 (0.67, 1.37)	0.822	0.059	52.9	Random
≤ 7	3	159	0.91 (0.51, 1.62)	0.746	0.065	63.3	Random
Area							
North	2	252	0.94 (0.51, 1.75)	0.854	0.035	77.5	Random
Midland	4	311	1.51 (0.80, 2.86)	0.206	0.033	65.7	Random
South	3	330	0.76 (0.49, 1.16)	0.205	0.596	0.0	Fixed
Average age							
≥ 55	4	450	0.95 (0.55, 1.64)	0.859	0.230	30.3	Fixed
< 55	5	443	0.94 (0.65, 1.36)	0.751	0.019	66.0	Random

*NOS* Newcastle–Ottawa Scale

### Correlation of LC3 expression with overall survival (OS)

**Fig. 3:**
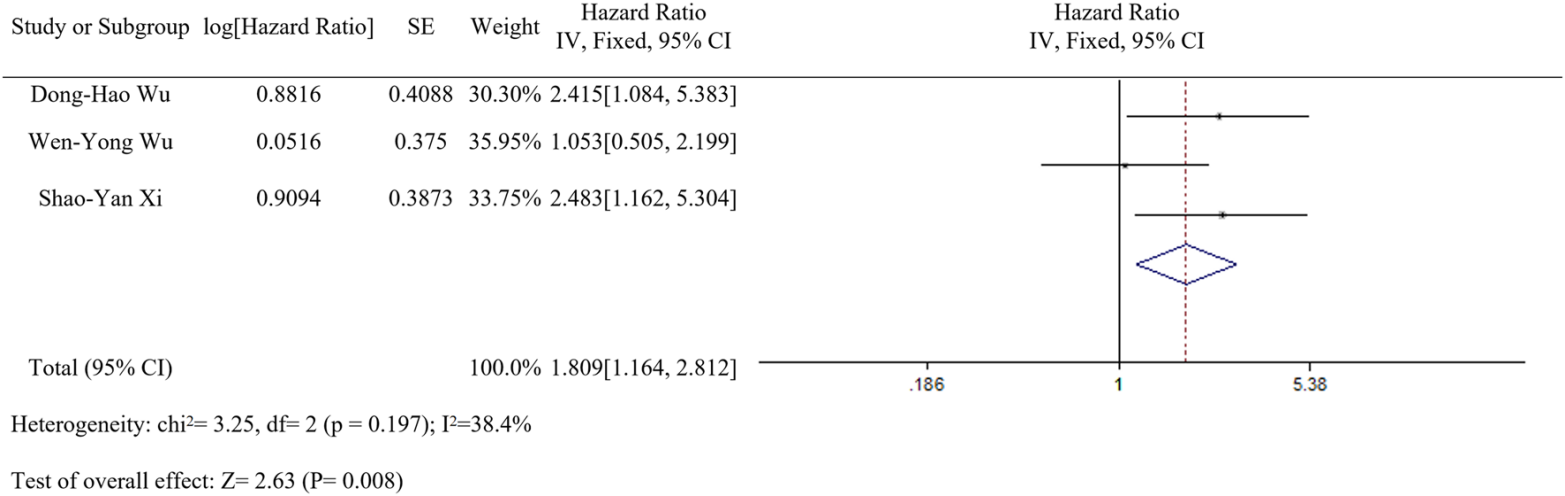
Forest plot of studies assessing the relationship between LC3 expression and OS in HCC patients

Based on meta-analysis, we evaluated the prognostic value of LC3 that high LC3 expression was correlation with the overall survival (HR 1.809, 95% CI [1.164, 2.812], *p* = 0.008, fixed effect) (Fig. 3).

### Publication bias

**Fig. 4:**
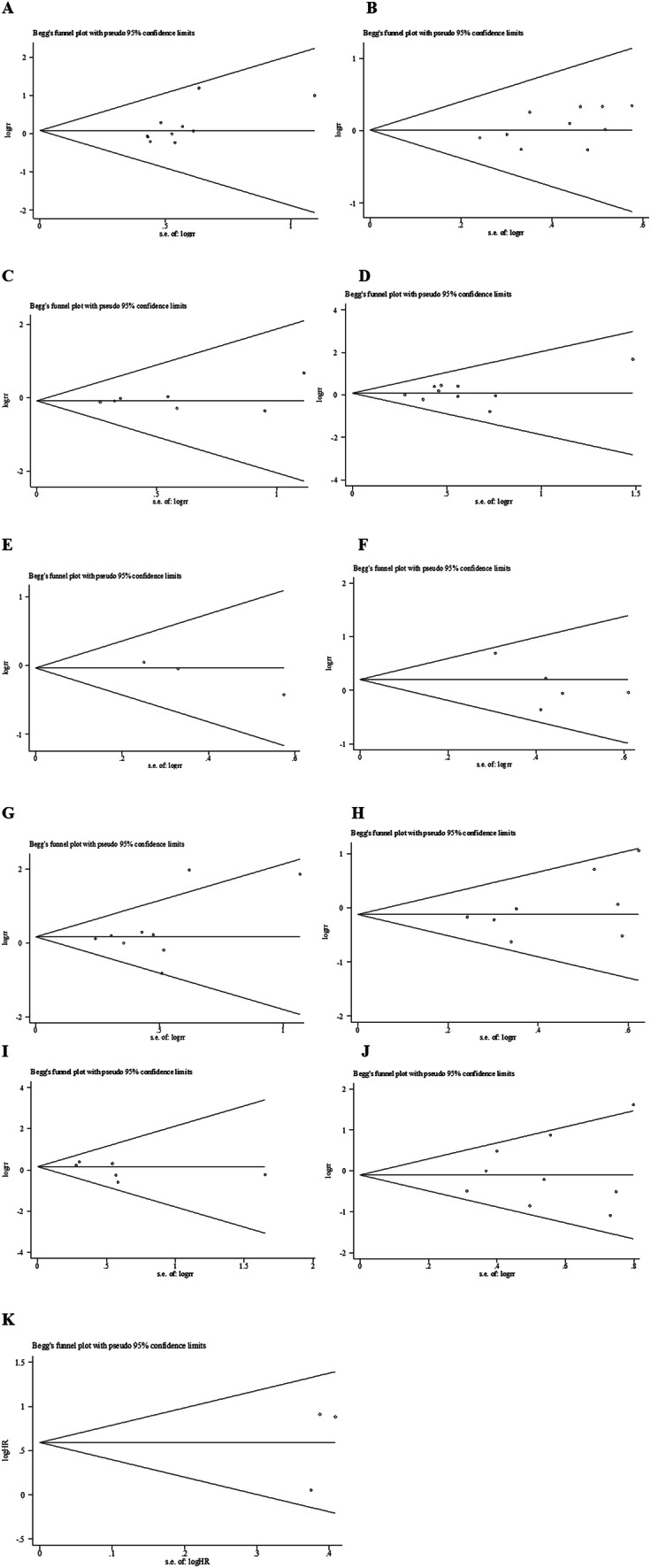
Funnel plot of studies assessing publication bias of the relationship between LC3 expression and** a** gender,** b** age,** c** liver cirrhosis,** d** HbsAg,** e** tumor number,** f** AFP,** g** tumor size,** h** TNM stage,** i** vascular invasion,** j** histological grade

As shown in Fig. 4, the Begg’s test was used to evaluate potential publication bias. No publication bias was confirmed to exist in gender (*p* = 0.074), age (*p* = 0.474), liver cirrhosis (*p* = 0.548), HBsAg (*p* = 0.592), number of tumors (*p* = 0.296), size of tumors (*p* = 0.466), TNM stage (*p* = 0.266), AFP (*p* = 0.806), OS (*p* = 0.602), vascular invasion (*p* = 0.133), histological grade (*p* = 0.348). We conduct a sensitivity analysis by excluding one study in turn. Interestingly, heterogeneity of histological grade declined from *I*^2^ = 50.3 to *I*^2^ = 37.6 after removing the article from Zhao et al. (2016).

## Discussion

Autophagy plays a crucial role in immune responses, cellular metabolism, apoptosis, and cell death (Choi et al. 2013). It has been reported that autophagy remains multiple roles in tumor, it may promote tumor progression and also can inhibit tumor transformation (Morselli et al. 2009). The attention of the relationship between autophagy and cancer have been increased. Recent studies have focused on evaluating the clinicopathological and prognostic values of autophagy-related markers in cancer revealed their clinical significances. LC3 is identified autophagy-related gene that plays a crucial role in the process of tumorgenesis (Galais et al. 2019). Currently, the role of LC3 in HCC is controversial reported. Therefore, we performed this meta-analysis to evaluating clinicopathological and prognostic values of LC3 in HCC.

In this meta-analysis, 949 patients from 10 studies were performed. Positive LC3 expression is related to tumor size but not to gender, age, number of tumors, HBsAg, liver cirrhosis, TNM stage, AFP, vascular invasion and histological grade. The relationship between LC3 expression and tumor size was explored, using 887 patients from 9 articles. Eight of the nine studies showed that LC3 expression was not associated with tumor size, but the pooled results showed a positive correlation between LC3 expression and tumor size. Tumor growth can cause the ischemic and hypoxic environment, LC3 may alleviate this state and enhance the environmental viability of tumor. The increase in tumor diameter induces a sustained increase in LC3 levels in tumor cells, which in turn induces autophagic recycle, provides nutrients for the tumor cells, and promotes tumor proliferation. Thus, this result indicates that LC3 may plays an important role in the occurrence and development of HCC. Moreover, our data indicated that high LC3 expression level is related to the overall survival in HCC, which also confirmed by Wu et al. (2014a) and Lee et al. (2013) studies. Therefore, the change of LC3 level may be related to the occurrence, evolution and poor prognosis of tumor, suggesting that the expression of LC3 is closely related to the occurrence and development of HCC.

This study has been the first comprehensive and systematic meta-analysis exploring the relationship between LC3 and its clinicopathological fators in HCC. Recent reports show that the combination of other related genes can improve clinical predictive value. Zhao et al. (2017) have found that beclin1 and LC3 can be potential prognostic markers in retrospective studies of ovarian cancer. Wu et al. (2018) have demonstrated that ULK1 combined with LC3B would improve prognosis assessment of the HCC patients and so on. Thus, LC3 can also be combined with other related genes for clinical diagnosis.

Significant heterogeneity with the correlation of LC3 expression and histological grade or tumor size was determined in this analysis. Sensitivity and subgroup analysis were performed to explore the sources of the heterogeneities. Subgroup analysis based on NOS showed that LC3 expression was uncorrelated with histological grade of HCC patients. The heterogeneity existed in subgroup of tumor size (*n* ≤ 7). The subgroups were classified by sample size, LC3 expression was related to tumor size (*n* > 100) but not to histological grade. On the basis of the area, heterogeneity was both in midland subgroup of tumor size and histological grade. Subgroup analysis based on mean age, heterogeneity was observed in tumor size subgroup (age ≥ 55) and histological grade subgroup (age < 55). This suggests that the reasons leading to the heterogeneity have many different characteristics. First, the detection methods and publication years are different. Second, IHC, a semi-quantitative assessment method, which is affected by many factors such as the concentration and incubation time of the antibody and diaminobezidin, the quality of the antibody, and so on, some of which are based on individual manners. This may be a potential factor for the occurrence of heterogeneity. Third, diverse sample sources exist in each study. Finally, lack of eligible articles for this meta-analysis. Therefore, more high-quality and large-scale studies on the relationship of LC3 and HCC should be performed in the future.

## Limitations of the study

Our meta-analysis has some limitations including: (1) the number of included articles was less, more high-quality studies need to be included; (2) included studies have different cut-off values for IHC scores, this may lead to a potential heterogeneity. The exploration of heterogeneity may have been inadequate, due to the limited variables collected from included studies; (3) of the included studies, nine were from China and one was from Korea, studies from other regions were not available; (4) some individual research data could not be obtained, and the analysis of the prognostic role of LC3 included only three studies, the research volume was relatively small.

## Conclusions

In summary, our meta-analysis indicated that positive LC3 is only positively correlated with tumor size in clinicopathological features of HCC. LC3 can be used a prognostic marker in HCC and combined with other related genes may improve clinical value. However, these results need to be confirmed by more large sample size and high-quality articles.
